# Pre-hospital healthcare for hyperemesis gravidarum: a cross-sectional analysis of baseline data from the SUKK-P study

**DOI:** 10.1186/s12884-026-09468-5

**Published:** 2026-06-18

**Authors:** Hilde Erdal, Jone Trovik, Kristine Heitmann, Andrea Negård, Anja Døssland Holstad, Katherine Tønnesen, Ingunn Krakeli, Lone Holst

**Affiliations:** 1 Centre for Pharmacy, Department of global public health and primary care, University of Bergen, Bergen, Norway; 2 Department of Obstetrics and Gynecology, Haukeland University Hospital, Bergen, Norway; 3 Department of Clinical Science, University of Bergen, Bergen, Norway; 4 Regional pharmacovigilance and medicines information center, Department of Medical Biochemistry and Pharmacology, Haukeland University Hospital, Bergen, Norway; 5 Vestre Viken Hospital Trust, Drammen Hospital, Drammen, Norway; 6 Innlandet Hospital Trust, Gjøvik Hospital, Gjøvik, Norway; 7 Telemark Hospital Trust, Skien, Norway; 8 St. Olavs Hospital, Trondheim University Hospital, Trondheim, Norway

**Keywords:** Hyperemesis gravidarum, Morning sickness, Pregnancy, Antiemetics, Ondansetron, Metoclopramide, Prochlorperazine, Meclizine, Promethazine

## Abstract

**Background:**

Women suffering fromhyperemesis gravidarum need timely and appropriate treatment to alleviate symptoms and prevent complications to the mother and fetus.

**Aim:**

The aim of this study was to identify and analyze treatment given prior to hospitalization to women included in the SUKK-P study.

**Methods:**

This study presents cross-sectional analyses at enrollment in the prospective SUKK-P cohort study of hyperemesis gravidarum (HG) treatment. Women hospitalized for HG were included from 11 departments within Norway between February 2021 and December 2023. Self-reported symptom severity and its impact on daily life at inclusion, treatment and health care received prior to hospitalization were collected by survey and chart review.

**Results:**

The majority (89%) of the 214 women included in SUKK-P had sought health care for nausea and vomiting during pregnancy (NVP)/HG prior to hospitalization. Although 88% had used one or more antiemetics prior to hospitalization, 42% were on sick leave for nausea prior to being offered antiemetic treatment. The median PUQE-score at hospitalization was 14 (interquartile range (IQR) 12–15) with a median wellbeing score of 2 (IQR 1–3). The participants reported severe impact on daily life in terms of reduced ability to perform household chores, care for children, and socialize. Nearly all (99%) were feeling low or depressed to some degree, and nine of ten reported that the nausea impacted the relationship with their partners. Half of the women reported having thoughts about terminating the pregnancy. One or more additional symptoms were reported by 97% with headache and hopelessness or feeling low as the most frequent, closely followed by difficulty sleeping, acid reflux, and constipation.

**Conclusions:**

Women with hyperemesis gravidarum describe high level of distress, still there is a delay in being provided antiemetics as 42% reported provision of sick leave as first healthcare measure.

**Supplementary Information:**

The online version contains supplementary material available at 10.1186/s12884-026-09468-5.

## Background

Nausea and vomiting in pregnancy (NVP) occurs to some degree in around 70% of pregnant people and is usually limited to the first trimester [1]. Hyperemesis gravidarum (HG) is a debilitating complication defined by persistent severe nausea and/or vomiting where The symptoms start early in pregnancy, before week 16, and the affected patients experience difficulty eating and/or drinking normally [2]. HG occurs in around 1–3% of pregnancies and may require hospitalization to correct dehydration, electrolyte imbalances, and malnutrition, as well as medication to manage nausea and vomiting [1].

Unless carefully managed, women with HG are at risk of severe maternal and fetal complications, such as anemia, preeclampsia, eclampsia, thromboembolism, Wernicke’s encephalopathy, electrolyte imbalance, preterm birth, induced labor, and caesarian section [1, 3, 4]. Further, a fatal consequence of HG is termination of an otherwise welcome pregnancy [5, 6]. Suffering from HG has a severe impact on the women’s ability to perform daily activity and on their mental health, illustrated by the occurrence of suicidal ideation, post-traumatic stress disorder following the pregnancy, and reluctance towards getting pregnant again [5–7]. HG is associated with a history of migraine, sleeping difficulties, excessive salivation, and NVP can be worsened by acid reflux [8–11]. Although these symptoms may impact the patients’ wellbeing, the occurrence among HG-patients is scarcely investigated.

NVP is among the most common pregnancy complaints and affects more than seven in ten women to some degree [12]. While physicians tend to focus on normalizing nausea and vomiting during pregnancy, women with HG can interpret this as trivialization of their severe symptoms [13]. After the thalidomide tragedy, many have been reluctant to prescribe and use medication for NVP [1]. For women hospitalized for HG at a Norwegian hospital, pre-hospital use of antiemetics was recorded in the patient charts of half (50%) in a previous study [14]. In a study from the US, 38% had pre-hospital prescriptions for antiemetics [15]. Several studies have shown that a high proportion of women being admitted for HG have not used antiemetic medication prior to hospitalization both in Norway and internationally. As milder nausea is generally more responsive to treatment, earlier treatment initiation may prevent progression to HG [16–18].

SUKK-P is a prospective, longitudinal cohort study in women hospitalized for treatment of HG, conducted to explore the provided care, describe symptom severity and duration, as well as impact of HG on daily life functioning. In addition, SUKK-P aims at exploring associations between maternal symptoms and characteristics and severity of HG to enable identification and early treatment initiation in women with risk of developing severe HG. This study describes the severity of symptoms and impact of HG on daily living in the SUKK-P study population at inclusion and explores healthcare and treatment provided in primary care for patients later hospitalized for HG treatment.

## Methods

This study presents findings of prospectively reported symptoms and cross-sectional analyses of baseline data from the SUKK-P longitudinal cohort study including data on the first hospitalization for HG.

### Participants

The target population was women hospitalized for treatment of hyperemesis gravidarum, defined as a diagnosis of O21.0 or O21.1 according to ICD-10. Inclusion criteria were age 18 or above, and the ability to complete questionnaires in Norwegian. Women hospitalized primarily for treatment of other conditions or with other severe disease were excluded.

### Setting

**Fig. 1:**
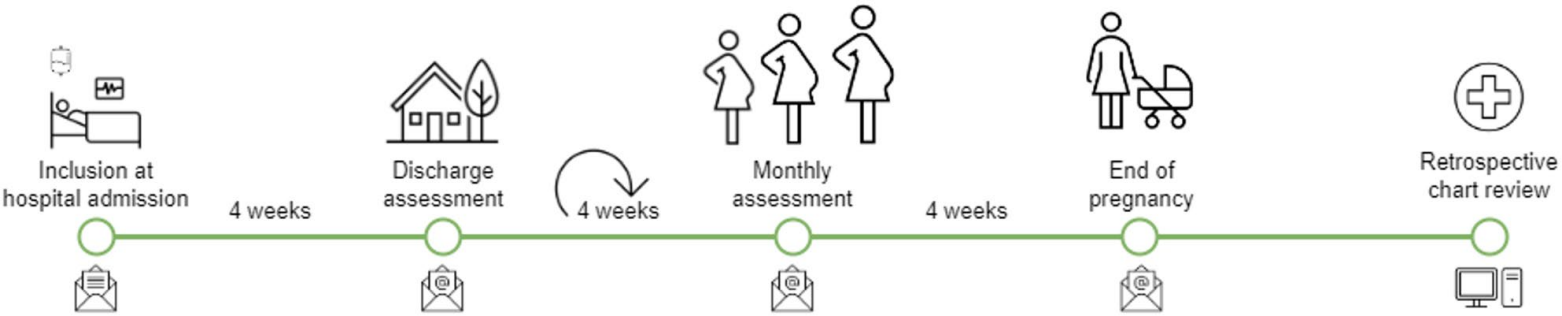
Data collection in SUKK-P prospective study of hyperemesis treatment

Potential participants were invited to SUKK-P by the department staff while hospitalized. In total, nine gynecological departments and two municipal in-patient facilities across Norway, including university and local hospitals, participated and distributed study material to eligible patients. Figure 1 illustrates the data collection process in SUKK-P and an overview of the data collected at baseline. The overall recruitment period was between February 18th 2021 and December 31st 2023. Details on departments and recruitment periods are provided in Supplementary Table 1. 

### Measurements

Self-reported data on maternal and pregnancy characteristics, symptoms, impact on daily activities, and healthcare was collected at the baseline questionnaire in SUKK-P (English translation provided in Supplementary file 1). Symptom severity was assessed using PUQE-24 score, which has been translated to and validated in Norwegian [19, 20]. Impact on daily life was explored in five questions invented for this study inspired by and adapted from previous studies [21, 22]; ability to perform daily household chores, care for children, live a social life, maintain the relationship with their partner, ability to work, feeling low or depressed, and ability to eat and drink normally were ranked on a five-point Likert scale ranging from not at all to very much. The impact on ability to eat and drink normally was included for participants recruited in 2023 as inability to eat and/or drink normally are included in the Windsor definition for HG.^2^ Furthermore, data on maternal and gestational characteristics and treatment provided at hospital were collected by retrospective review of hospital medical charts. The participants were classified as native Norwegian if Norwegian or Sami was reported as their first language.

### Statistics

Normally distributed data was presented as means with corresponding standard deviation (SD), while others as median with corresponding interquartile range (IQR). The responses from the paper baseline questionnaire were transferred to REDCap (Research Electronic Data Capture), a secure, web-based software platform hosted at the University of Bergen [23, 24]. Logistic regression was used to investigate differences in seeking healthcare and receiving pre-hospital antiemetic treatment between native Norwegian and non-native Norwegian participants adjusted for maternal age, body mass index, and whether the woman was pregnant for the first time. Similarly, associations between reported PUQE-score, other symptoms and thoughts of pregnancy termination, and reported symptoms and use of pre-hospital antiemetic medication were investigated. Crude and adjusted odds ratio were presented with corresponding confidence interval and a statistical significance threshold was set at 95%. Data were analyzed using Stata (StataCorp. 2023. Stata Statistical Software: Release 18. College Station, TX: StataCorp LLC).

### Ethics

The SUKK-P study has been approved by the Norwegian Regional Committees for Medical and Health Research Ethics (REK Sør-Øst 2020/133791) and the Data Protection Officers at all participating institutions prior to study start. All participants signed informed consent.

## Results

Of a total of 373 women hospitalized for HG invited to participate, 214 (57%) were included in SUKK-P (Supplementary Table 1). One in three (*n* = 66, 31%) of the participants were pregnant for the first time. Of the women who were not pregnant for the first time, 99 (67%) self-reported at least one prior pregnancy with HG. Of these, 10 women (10%) reported having performed termination of a previous pregnancy due to HG. Comparably, a history of HG was documented in the hospital chart of 67 women, leading to a total of 107 (72%) participants having indication of a history of HG. Further characteristics are presented in Table 1.

**Table 1:** Descriptive characteristics of 214 participants with hyperemesis gravidarum (HG) in the SUKK-P study

Maternal and gestational characteristics at hospitalization for HG	
Age in years, mean (standard deviation (SD))	29.5 (5.3)
Norwegian or Sami first language, number (%)	161 (75)
Pre-pregnancy body mass index in kg/m^2^, mean (SD)^a^	25.6 (4.6)
Gravidity, median (inter-quartile range (IQR))	2 (1; 3)
Parity, median (IQR)	1 (0; 1)
Number of women with previous pregnancy with HG, n (%)^b^	107 (74)
PUQE-score, median (IQR)^c^	14 (12; 15)
Wellbeing score, median (IQR)^d^	2 (1; 3)
Weight loss, in % of pre-pregnancy weight, mean (SD)^e^	3.3 (4.8)
Gestational age when nausea started, in days, median (IQR)^f,g^	40 (35; 45)
Gestational age at hospitalization, in days, median (IQR)^f,h^	63 (55; 79)
Self-reported estimate of time between first onset of nausea and vomiting and experiencing need for medical treatment,n (%)^i^:	
Less than one week	82 (39)
One to two weeks	67 (31)
Two to four weeks	43 (20)
More than four weeks	21 (10)

^a^*n*=190, ^b^either self-reported or from hospital chart in % of *n*=144 with one or more previous pregnancies, ^c^*n*=207, ^d^*n*=97, ^e^*n*=196, ^f^gestational age calculated from due date determined at routine ultrasound examination in gestational week 18-20. ^g^*n*=171, ^h^*n*=185, ^i^Based on the question: “How quickly did the nausea worsen from the first signs of nausea until you were so sick that you needed treatment from a doctor”, *n*=213

**Fig. 2:**
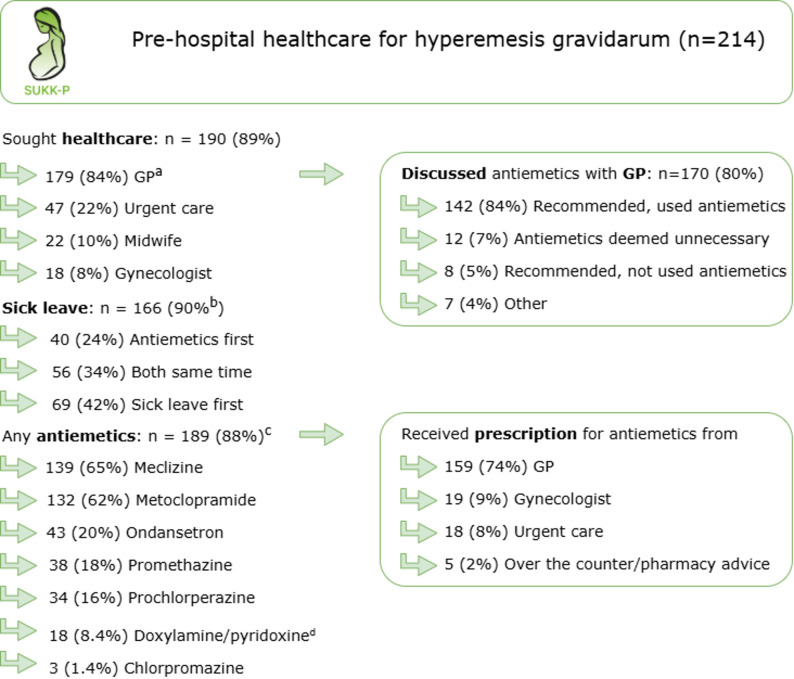
Self-reported pre-hospital healthcare and antiemetic treatment at enrollment in SUKK-P (*n*=214). ^a^GP=General practitioner, ^b^of *n*=185, *n*=29 (14%) not currently working. ^c^Use of pre-hospital antiemetics either self-reported or from hospital chart, ^d^Doxylamine/pyridoxine marketed for NVP in Norway in May 2022 and was used by 30% of participants recruited in 2023. The summarized percentages exceed 100% when several alternatives may apply

Self-reported healthcare in primary care prior to hospitalization is illustrated in Fig. 2. Overall, pre-hospital use of one or more antiemetic medication either from chart or self-reported was recorded for 88% of the participants. Self-reported pre-hospital use of antiemetics (85%) was slightly higher compared to information from the patient medication chart (82%), probably partially accounted for by five participants (2.3%) reporting buying or being recommended over-the-counter antihistamines for NVP at a pharmacy. There was no significant difference between Norwegian participants (75%) and those with other primary languages in the odds of seeking healthcare or receiving pre-hospital antiemetics (*p* = 0.249 and *p* = 0.691, respectively), and adjustment for age, maternal body mass index, and primigravida did not change the results (Supplementary Table 2).

Of all the participants, 14 (3.2%) answered that they chose not to use recommended or prescribed antiemetic treatment prior to hospitalization due to concerns for fetal safety while three (1.4%) refrained for maternal safety concerns. 

**Fig. 3:**
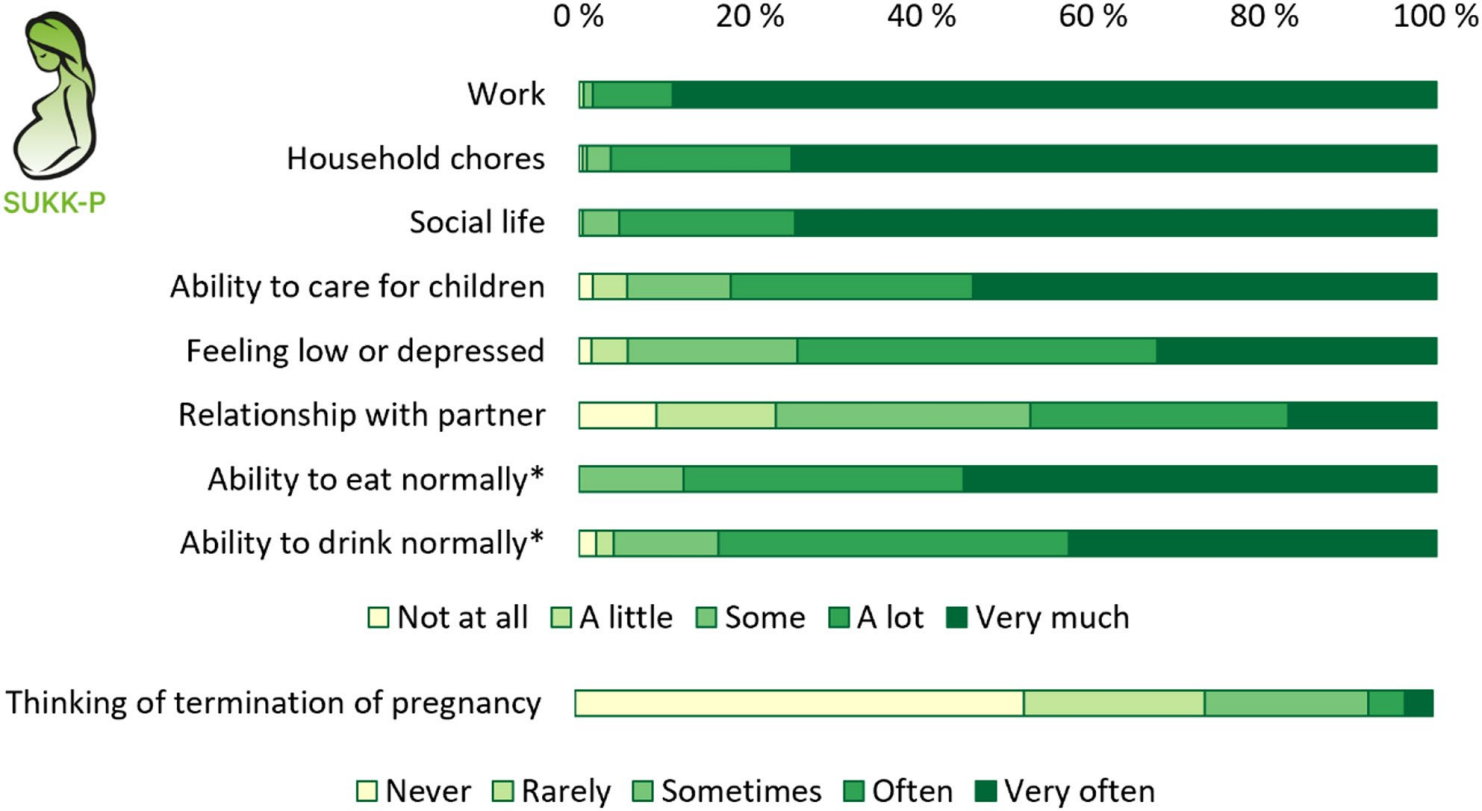
Impact of nausea and vomiting on activities of daily living and thoughts of termination of pregnancy in the past week ranked on a 5-point Likert scale at enrollment in SUKK-P prospective study of hyperemesis treatment, *n* = 214. *n = 49 (only assessed by women recruited in 2023)

**Fig. 4:**
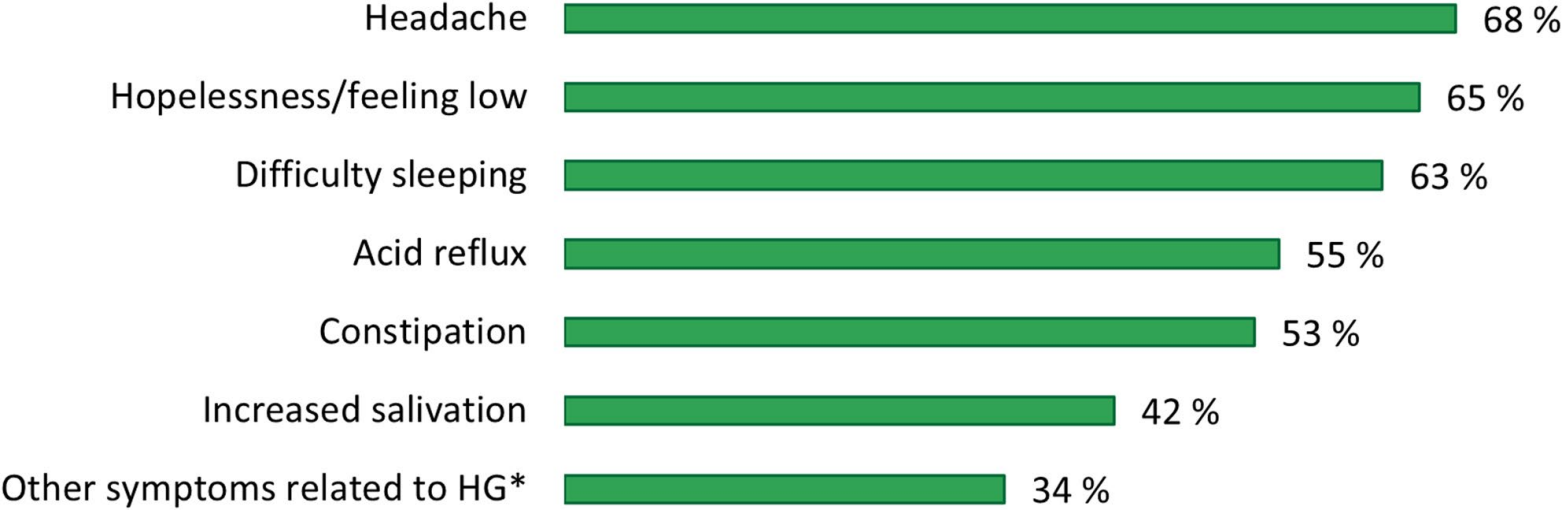
Frequency of selected symptoms experienced by women hospitalized for hyperemesis gravidarum at inclusion in SUKK-P, *n* = 214. *The most frequently mentioned symptoms reported in free text were fatigue, dizziness, diarrhea, and stomachache

The median number of days between hospitalization and completion of the questionnaire was 1 day (IQR 1–2). At the time of enrollment, the self-reported PUQE-score was 13 (IQR 12–15) with a corresponding wellness score of 3 (IQR 2–4). Figure 3 shows the participants’ assessment of the impact of HG on their daily living activities as well as thoughts of termination of pregnancy (frequencies provided in Supplementary Table 3). In addition to suffering from severe nausea and vomiting, nearly all (97%) of the participants reported one or more other symptom at enrollment (Fig. 4). In total, 102 participants (48%) reported having had thoughts of terminating the pregnancy (Fig. 4).

The odds of thinking of termination of pregnancy was increased more than threefold in women reporting feeling low or feeling hopelessness (OR 3.65, 95% confidence interval 1.86–7.17, *p* < 0.001) (Supplementary Table 2). There was no significant association between thoughts of termination and an increase in PUQE-score at hospitalization (OR 1.04, 95% confidence interval 0.90–1.20, *p* = 0.628), or thoughts of termination and the other symptoms, except for a reduced odds in women reporting acid reflux (OR 0.36, 95% confidence interval 0.19–0.68, *p* = 0.001) (Supplementary Table 2). Decreasing wellbeing score was significantly associated with the odds of thinking about pregnancy termination (OR 0.81, 95% confidence interval 0.70–0.94, *p* = 0.007). In those reporting constipation, the odds of using ondansetron was significantly increased (OR 5.54, 95% confidence interval 2.26–13.6 *p* < 0.001) (Supplementary Table 2). No further associations were found between symptoms and antiemetic medication (data not shown). 

## Discussion

In this cross-sectional analysis of the prospective cohort study SUKK-P, we found that the vast majority (89%) of the women hospitalized for HG had sought healthcare and that most (88%) had tried one or more antiemetic medications prior to hospitalization. Our findings illustrate the debilitating impact of HG on several activities related to daily living, and that 90% had been on sick leave. Further, the patients frequently experienced headaches, feeling low or experiencing hopelessness, sleeping difficulties, acid reflux and constipation adds to the massive impact of HG on all aspects of daily living, as well as the importance of addressing and managing symptoms beyond nausea and vomiting.

Our findings highlight the early onset of and rapid deterioration of symptoms requiring medical intervention. Surprisingly, as many as four in ten reported a need for treatment within a week of symptom debut. Together with the finding that hospitalization occurred at a median of 23 days after symptom debut, the treatment window for management in primary care is limited for many with severe HG. Further, we found that approximately nine in ten sought healthcare in primary care prior to hospitalization. Of the nearly 90% on sick leave, only six of ten were prescribed antiemetic treatment prior to or simultaneously with sick leave, thus four in ten were assessed as too sick to work without receiving pharmacological treatment for NVP. This aligns with a qualitative study where Norwegian primary care physicians described first considering sick leave and dietary and lifestyle changes for NVP, while being reluctant towards prescribing antiemetics [13]. Similarly, UK primary care physicians have described a lack of confidence in prescribing recommended antiemetics for NVP and HG [25]. Nonetheless, nearly nine of ten received antiemetic treatment before hospitalization, primarily prescribed by primary care physicians, which indicates repeated visits. Our findings suggest a delay in pharmacological treatment initiation when women developing severe HG seek healthcare for worsening NVP in primary care, further illustrated by one in five being in contact with urgent care. Future studies should investigate whether earlier pharmacological treatment initiation might prevent hospitalization in some women with HG.

Among those who had been pregnant before, 72% had suffered from HG in one or more earlier pregnancies and 10% of these reported having performed pregnancy termination because of HG. Our findings are in line with previous findings of high recurrence rates of HG,^6^ and previous studies showing high rates of termination of pregnancy among HG sufferers [14]. Thinking about pregnancy termination (either seldom, sometimes, often, or very often) in this pregnancy was reported by nearly half of the participants. Having these thoughts were not directly associated with PUQE-score, which may be explained by the PUQE-24 score not directly reflecting symptom load in women with severe HG. The ceiling effect of 15 points includes a large proportion of the patients. Additionally, those who vomit excessively may have a deceptively low PUQE-score as they might not experience retching at all, leading to a maximum PUQE-score of 11 points. Thinking about pregnancy termination was, however, significantly associated with feeling low or experiencing hopelessness as well as reporting low wellbeing at the PUQE-24 score. Similarly, around half of the participants in a large survey from the UK considered pregnancy termination, and termination was associated with lower perception of quality of care and suicidal ideation [26]. Qualitatively, the patients in the UK study described a massive impact on daily living, feelings of hopelessness and desperation, and that pregnancy termination was chosen as an alternative to suicide [5]. This underscores the importance of addressing and managing the maternal health impact of HG as 65% of the participants in our study reported feeling low or having thoughts of hopelessness.

Interestingly, reporting acid reflux was inversely correlated with thinking of termination of pregnancy. We have not found this previously reported, and it may merit future investigation into possible clinical differences in women with different presentations of HG. Acid reflux is common in pregnancy due to hormone-induced relaxation of smooth musculature, and can increase in severity and frequency during the pregnancy and may aggravate symptoms of NVP [9]. Despite being as early in pregnancy as a median of 7 weeks, 55% of the participants reported acid reflux at inclusion.

A general reluctance towards using medicines in pregnancy developed after the Thalidomide tragedy [27], and women with NVP have described concerns with using medication in pregnancy [13, 28] To explore whether this affected the willingness to use antiemetic medication in this cohort, we specifically asked if safety concerns in pregnancy had led to deliberate non-compliance. Reassuringly, only 13 women (8%) reported not using recommended medication for fetal safety concerns, while 3 (2%) stated concern for their own health. This suggests that the majority were provided with sufficient information about the medicines, and that the recommended treatment was perceived to outweigh any potential risks.

### Strength and limitations

We present a large cross-sectional study of women hospitalized for treatment of HG from a variety of departments across Norway, including university hospitals, local hospitals, and municipal in-patient facilities. Prospectively collected patient-reported measures of symptom severity, impact on daily activities, health care, and treatment adds information not available from registries or patient charts without risk of recall bias. A further strength of the study is the addition of chart review of medical records to provide treatment data in detail and with limited bias and risk of misclassification. However, questionnaires in Norwegian introduces a selection bias, limiting the diversity of the eligible study population. Further, only 57% of those invited were included. Reasons for declining participation have not been collected, but challenges in participating in a prospective study while severely ill may limit the representativeness of the findings to the most severely affected patients. A validated instrument for measuring the impact of HG on daily living was not found. Consequently, we developed a set of questions to explore the impact of HG on selected aspects of daily living and a selection of additional symptoms was suggested based on our clinical experience and previous literature,^8, 9^ a limitation of which is that the instrument has not been validated in the study population. A strength of the study is that representatives from the patient organization Hyperemesis Norge participated in developing and testing the questionnaires and consulted on the study protocol.

## Conclusion

Our findings provide further evidence of the debilitating impact suffering from severe HG has on women’s wellbeing and their ability to participate in daily activities. Nearly half of the participants in SUKK-P reported experiencing thoughts of terminating the pregnancy, and many suffered from feeling low/hopelessness, headache, and sleeping difficulties in addition to nausea and vomiting.

Although prehospital treatment with antiemetics was reported by nine of ten in this study, around half of the women had been on sick leave prior to any pharmacological treatment, suggesting a gap in the care pathway. Improvements may enable timely treatment initiation for women presenting with NVP in primary care. Further efforts should be made to ensure that women at risk of developing severe NVP and HG are identified and provided with appropriate treatment and to investigate whether earlier treatment initiation prevents hospitalization. 

## Supplementary Information

Supplementary Material 1. English translation of the SUKK-P baseline questionnaire

Supplementary Material: Table S1. Overview of study sites, recruitment period, and the number of patients invited to and included in SUKK-P prospective study of hyperemesis treatment. Table S2 Logarithmic regression analysis of group associations in seeking healthcare, pre-hospital antiemetics, and between symptoms and thinking about pregnancy termination. Table S3 Self-reported impact of hyperemesis gravidarum on daily life functioning at enrollment in SUKK-P ranked on a 5-point Likert scale, *n*=214.

## Data Availability

The data used in the current study is not publicly available due to sensitive personal information. Anonymized aggregated data are available from the corresponding author upon reasonable request.

## Abbreviations

HG: Hyperemesis gravidarum
NVP: Nausea and vomiting during pregnancy
GP: General practitioner
PUQE: Pregnancy Unique Quantification of Emesis
