# Supplementary material for: Pre-hospital healthcare for hyperemesis gravidarum: a cross-sectional analysis of baseline data from the SUKK-P study
Source: BMC Pregnancy Childbirth. 2026 Jun 18;26:724. doi: 10.1186/s12884-026-09468-5 (PMC13343708; doi:10.1186/s12884-026-09468-5)
Supplement: Supplementary file 1 — Supplementary Material 1. English translation of the SUKK-P baseline questionnaire [file 12884_2026_9468_MOESM1_ESM.docx]

| **SUKK-P**  **Prospective study of treatment of hyperemesis gravidarum** |  |
| --- | --- |

Questionnaire number 1

1. Today’s date:

|  |
| --- |

2. Is Norwegian or Sami your mother tongue?

❑ Yes. Proceed to the next page.

❑ No

3. If no, which continent does your mother tongue origin from?

❑ The Nordic countries

❑ Other European countries

❑ Asia

❑ Oceania

❑ Africa

❑ South America

❑ North America

4. How many years have you lived in Norway?

|  |
| --- |

5. Date of the first day of the last menstrual period?

|  |
| --- |

6. How much did you weigh before you got pregnant (in kilos)?

|  |
| --- |

7. What is your current weight (in kilos)?

|  |
| --- |

8. When did the nausea start? Date:

|  |
| --- |

9. How quickly did the nausea worsen from the first signs of nausea until you were so sick that you needed treatment from a doctor?

❑ Less than a week

❑ One to two weeks

❑ Two to four weeks

❑ Gradually over more than four weeks

| Tick the answer that best describe your situation the last 24 hours prior to admission to hospital: |
| --- |

10. How many hours have you felt nauseous or sick to your stomach?

❑ Not at all

❑ ≤ 1 hour

❑ 2-3 hours

❑ 4-6 hours

❑ > 6 hours

11. How many times have you vomited?

❑ Not at all

❑ 1-2 times

❑ 3-4 times

❑ 5-6 times

❑ ≥ 7 times

12. How many times have you had retching or dry heaves without bringing anything up?

❑ Not at all

❑ 1-2 times

❑ 3-4 times

❑ 5-6 times

❑ ≥ 7 times

13. Assessment of wellbeing: On a scale of 0 to 10, how would you rate your wellbeing now; 0 = worst possible, 10 = as good as I felt before pregnancy.

| 0 | 1 | 2 | 3 | 4 | 5 | 6 | 7 | 8 | 9 | 10 |
| --- | --- | --- | --- | --- | --- | --- | --- | --- | --- | --- |
| ❑ | ❑ | ❑ | ❑ | ❑ | ❑ | ❑ | ❑ | ❑ | ❑ | ❑ |

14. Have you so far during this pregnancy been even more nauseous than you felt the last 24 hours before being admitted to hospital?

❑ Yes

❑ No

| How would you say that nausea and vomiting affects your daily life with regard to the following? Consider how it has been on average the last week. |
| --- |

15. To which degree does nausea affect daily activity related to household chores?

❑ Not at all

❑ To a small degree

❑ To some degree

❑ To a large degree

❑ To a very large degree

16. To which degree does nausea reduce your social activities?

❑ Not at all

❑ To a small degree

❑ To some degree

❑ To a large degree

❑ To a very large degree

17. To which degree does nausea negatively affect your relationship with your partner?

❑ Not at all

❑ To a small degree

❑ To some degree

❑ To a large degree

❑ To a very large degree

❑ I do not have a partner

18. To which degree does nausea affect your ability to care for your children?

❑ Not at all

❑ To a small degree

❑ To some degree

❑ To a large degree

❑ To a very large degree

❑ I do not have responsibility to care for children

How would you say that nausea and vomiting affects your daily life with regard to the following? Consider how it has been on average the last week.

19. To which degree does nausea reduce your capacity to do your work?

❑ Not at all

❑ To a small degree

❑ To some degree

❑ To a large degree

❑ To a very large degree

❑ I am not working

20. To which degree do you feel low or depressed because of nausea?

❑ Not at all

❑ To a small degree

❑ To some degree

❑ To a large degree

❑ To a very large degree

21. If you have a job; have you been on sick leave so far in this pregnancy because of nausea?

❑ Yes

❑ No. Proceed to question 26.

❑ I do not have a job. Proceed to question 26.

22. If yes, have you been on full or graded sick leave?

❑ Full sick-leave

❑ Graded sick leave

❑ Both

23. Approximately, for how long have you been on sick-leave so far in this pregnancy (number of weeks with any grade of sick-leave because of nausea)?

|  |
| --- |

24. Who suggested sick leave?

❑ The doctor

❑ Myself

❑ Others (specify below):

|  |
| --- |

25. If you have been on sick-leave because of nausea, did you receive sick-leave before you were prescribed treatment with antiemetics?

❑ Yes

❑ No, I was first prescribed antiemetics and was later put on sick leave

❑ No, I was put on sick leave at the same time as I was prescribed antiemetics

26. Did you seek health care for nausea and vomiting before you were so sick that you had to be admitted to hospital? For instance, contacted your general practitioner, midwife or other health care services?

❑ Yes

❑ N0

27. If no, describe why you did not seek health care before you were so sick that you had to be admitted to hospital:

|  |
| --- |
|  |
|  |

28. If yes, who did you contact? You can tick off several options.

❑ My general practitioner

❑ Gynecologist

❑ Midwife

❑ Doctor at hospital

❑ Doctor at emergency room

❑ Others (specify below):

|  |
| --- |

29. Did you discuss treatment with antiemetics with your GP prior to admission to hospital?

❑ Yes

❑ No

30. If not, why?

❑ I have not been in contact with my GP before I was admitted acutely

❑ Neither my GP nor I brought up the topic

❑ Other, or something you wish to specify below

31. If yes, which option is most fitting?

❑ My doctor and I agreed that treatment was not necessary

❑ I brought up the subject, but my doctor said it was not necessary

❑ My doctor brought up the subject, but I felt it was not necessary

❑ I was recommended/prescribed antiemetic medication and have used this prior to admission

❑ I was recommended/prescribed antiemetic medication but chose not to use it

❑ Other, or something you wish to elaborate below

32. Specify or elaborate here:

|  |
| --- |
|  |
|  |

33. If you have been prescribed antiemetics from your doctor, but have not used them, why did you choose not to use the medication?

❑ Because I was concerned about the safety for the health of the child

❑ Because I was concerned about the safety of my own health

❑ Other (specify below)

|  |
| --- |
|  |

34. If you have been prescribed antiemetics prior to hospital admission, which of the following medicines? Tick off all of the medicines you have been prescribed before hospital.

❑ Meclizine

❑ Metoclopramide

❑ Ondansetron

❑ Prochlorperazine

❑ Promethazine

❑ Chlorpromazine

❑ Other antiemetics (specify below):

|  |
| --- |

35. If yes, who prescribed the medicines? You can tick off several options.

❑ My GP

❑ Gynaecologist

❑ Emergency room doctor

❑ Others (specify below):

|  |
| --- |

36. Have you experienced any of these symptoms so far in this pregnancy, in addition to nausea?

❑ Acid reflux

❑ Increased saliva

❑ Constipation

❑ Headache

❑ Sleep disturbance

❑ Feeling depressed or a feeling of hopelessness

❑ Other symptoms related to hyperemesis? Specify below:

|  |
| --- |

❑ I have no other symptoms

37. Is this your first pregnancy?

❑ Yes. Proceed to the next page.

❑ No

38. How many times have you been pregnant before this pregnancy?

|  |
| --- |

39. Have you had hyperemesis in prior pregnancies?

❑ Yes

❑ No. Proceed to the next page.

40. In how many previous pregnancies have you had hyperemesis?

|  |
| --- |

41. When having hyperemesis in previous pregnancies, did you experience thoughts about ending the pregnancy (having an abortion) because of the nausea?

❑ Never

❑ Rarely

❑ Sometimes

❑ Often

❑ Very often

42. Have you ended a pregnancy (had an abortion) because of nausea and vomiting?

❑ Nei

❑ Ja

43. Have you so far in this pregnancy had thoughts about ending the pregnancy (having an abortion) because of hyperemesis?

❑ Never

❑ Rarely

❑ Sometimes

❑ Often

❑ Very often

44. Other comments or input to this study?

|  |
| --- |
|  |
|  |

| \|  \| \| --- \| \|  \| \|  \| |
| --- | --- | --- | --- |
