# Supplementary material for: Pre-hospital healthcare for hyperemesis gravidarum: a cross-sectional analysis of baseline data from the SUKK-P study
Source: BMC Pregnancy Childbirth. 2026 Jun 18;26:724. doi: 10.1186/s12884-026-09468-5 (PMC13343708; doi:10.1186/s12884-026-09468-5)
Supplement: Supplementary file 2 — Supplementary Material: Table S1. Overview of study sites, recruitment period, and the number of patients invited to and included in SUKK-P prospective study of hyperemesis treatment. Table S2 Logarithmic regression analysis of group associations in seeking healthcare, pre-hospital antiemetics, and between symptoms and thinking about pregnancy termination. Table S3 Self-reported impact of hyperemesis gravidarum on daily life functioning at enrollment in SUKK-P ranked on a 5-point Likert scale, n=214. [file 12884_2026_9468_MOESM2_ESM.docx]

**Supplementary material**

**Supplementary Table 1** Overview of study sites, recruitment period, and the number of patients invited to and included in SUKK-P prospective study of hyperemesis treatment.

|  | | **Patients^a^** | **Invited**  **n (%^b^)** | **Included**  **n (%^c^)** |
| --- | --- | --- | --- | --- |
| **Overall** | | **957** | **373 (39%)** | **214 (57)** |
|  | |  |  |  |
| **Hospital departments** | |  |  |  |
|  | Haukeland University Hospital  *18.Feb 2021-31. Dec 2023* | 290 | 132 (46) | 90 (68) |
|  | Bærum Hospital  *1. Nov 2021-31. Dec 2023* | 198 | 39 (20) | 24 (62) |
|  | Drammen Hospital  *1. Nov 2021-31. Dec 2023* | 94 | 29 (31) | 9 (31) |
|  | Lillehammer Hospital  *1. Jul 2021-31. Dec 2023* | 40 | 13 (33) | 11 (85) |
|  | Nordland Hospital, Bodø  *1. Jul 2021-31. Dec 2023* | 32 | 17 (53) | 11 (65) |
|  | St. Olavs Hospital  *1. Aug 2021-31. Dec 2023* | 121 | 52 (43) | 9 (17) |
|  | Gjøvik Hospital  *1. Jul 2021-31. Dec 2022* | 30 | 8 (27) | 4 (50) |
|  | Telemark Hospital, Skien  *1. Jul 2021-31. Dec 2022* | 42 | 12 (29) | 5 (42) |
|  | Stavanger University Hospital  *1. Sep 2021-31. Dec 2022* | NA | NA | 1 (NA) |
|  |  |  |  |  |
| **Municipal in-patient acute care units** | |  |  |  |
|  | Jæren  *1. Oct 2021-31. Dec 2023* | 71 | 37 (52) | 33 (89) |
|  | Stavanger  *1. Sep 2021-31. Dec 2023* | 39 | 34 (87) | 17 (50) |
| ^a^Patients hospitalized with an ICD-10 diagnosis of O21. ^b^Invitation rate in percent of patients hospitalized with HG. ^c^Inclusion rate in percent of patients invited to participate. | | | | |

<<**Supplementary table 2** Logarithmic regression analysis of group associations in seeking healthcare, pre-hospital antiemetics, and between symptoms and thinking about pregnancy termination.>>

|  |  |  |  |  |
| --- | --- | --- | --- | --- |
| **Sought healthcare** |  | **Odds ratio** | **Confidence interval (CI)** | **p-value** |
| Native Norwegian |  | Reference |  |  |
| Non-native Norwegian | Crude | 1.72 | 0.68-4.32 | 0.249 |
|  | Adjusted^a^ | 1.33 | 0.47-3.76 | 0.595 |
|  |  |  |  |  |
| **Pre-hospital antiemetics** |  | **Odds ratio** | **CI** | **p-value** |
| Native Norwegian |  | Reference |  |  |
| Non-native Norwegian | Crude | 1.21 | 0.48-3.08 | 0.691 |
|  | Adjusted^a^ | 1.38 | 0.52-3.68 | 0.514 |
|  |  |  |  |  |
| **Thougths of pregnancy termination** | | **Odds ratio** | **CI** | **p-value** |
| Headache | Crude | 0.95 | 0.53-1.69 | 0.863 |
|  | Adjusted^b^ | 0.95 | 0.51-1.79 | 0.882 |
|  |  |  |  |  |
| Hopelessness/feeling low | Crude | 3.46 | 1.89-6.36 | **<0.001** |
|  | Adjusted^b^ | 3.65 | 1.86-7.17 | **<0.001** |
|  |  |  |  |  |
| Difficulty sleeping | Crude | 0.79 | 0.46-1.38 | 0.417 |
|  | Adjusted^b^ | 0.73 | 0.40-1.36 | 0.323 |
|  |  |  |  |  |
| Acid reflux | Crude | 0.47 | 0.27-0.82 | **0.008** |
|  | Adjusted^b^ | 0.36 | 0.19-0.68 | **0.001** |
|  |  |  |  |  |
| Constipation | Crude | 1.59 | 0.93-2.73 | 0.093 |
|  | Adjusted^b^ | 1.42 | 0.78-2.58 | 0.245 |
|  |  |  |  |  |
| Increased salivation | Crude | 1.48 | 0.86-2.56 | 0.158 |
|  | Adjusted^b^ | 1.43 | 0.76-2.67 | 0.264 |
|  |  |  |  |  |
| PUQE-24 score at hospitalization (n=207) | Crude | 1.04 | 0.90-1.20 | 0.628 |
|  |  |  |  |  |
| PUQE-24 wellbeing score at inclusion (n=212) | Crude | 0.81 | 0.70-0.94 | 0.007 |
|  |  |  |  |  |
| **Constipation** |  | **Odds ratio** | **CI** | **p-value** |
| Meclizine |  | 1.14 | 0.61-2.13 | 0.691 |
| Metoclopramide |  | 1.04 | 0.58-1.89 | 0.890 |
| Ondansetron |  | 5.54 | 2.26-13.6 | **<0.001** |
| Promethazine |  | 2.09 | 0.94-4.62 | 0.070 |
| Prochlorperazine |  | 1.18 | 0.49-2.82 | 0.716 |
| Chlorpromazine |  | 0.13 | 0.01-1.99 | 0.142 |
| Doxylamine/pyridoxine |  | 1.56 | 0.55-4.41 | 0.404 |
| ^a^Adjusted for age, body mass index, and being pregnant for the first time or not. ^b^Adjusted for the other symptoms. | | | | |
|  |  |  |  |  |
|  |  |  |  |  |

**<<Supplementary Table 3** Self-reported impact of hyperemesis gravidarum on daily life functioning at enrollment in SUKK-P ranked on a 5-point Likert scale, n=214.>>

|  | Number (%) | | | | |
| --- | --- | --- | --- | --- | --- |
|  | Not at all | A little | Some | A lot | Very much |
| Work (n=183 working) | 0 (0) | 1 (0.5) | 2 (1) | 17 (9) | 163 (89) |
| Chores | 1 (0.5) | 1 (0.5) | 6 (3) | 45 (21) | 161 (75) |
| Social life | 0 (0) | 1 (0.5) | 9 (4) | 44 (21) | 160 (75) |
| Ability to care for children (n=124 with children) | 2 (2) | 5 (4) | 15 (12) | 35 (28) | 67 (54) |
| Feeling low or depressed | 3 (1) | 9 (4) | 42 (20) | 89 (42) | 69 (33) |
| Relationship with partner (n=209 with partner) | 19 (9) | 29 (14) | 62 (30) | 63 (30) | 36 (17) |
| Ability to eat normally* | 0 (0) | 0 (0) | 6 (12) | 16 (33) | 27 (55) |
| Ability to drink normally* | 1 (2) | 1 (2) | 6 (12) | 20 (41) | 21 (43) |
|  |  |  |  |  |  |
|  | Never | Rarely | Sometimes | Often | Very often |
| Thoughts of pregnancy termination | 112 (52) | 45 (21) | 41 (19) | 9 (4) | 7 (3) |
| *n=49 (only assessed by women recruited in 2023) | | | | | |
